# Supplementary material for: Artificial dual solid-electrolyte interfaces based on in situ organothiol transformation in lithium sulfur battery
Source: Nat Commun. 2021 May 28;12:3031. doi: 10.1038/s41467-021-23155-3 (PMC8163853; doi:10.1038/s41467-021-23155-3)
Supplement: Supplementary file 1 — Supplementary Information [file 41467_2021_23155_MOESM1_ESM.pdf]

Artificial dual solid-electrolyte interfaces based on in situ organothiol transformation in lithium sulfur battery

Wei Guo[1,4], Wanying Zhang[1,4], Yubing Si[1], Donghai Wang[2], Yongzhu Fu[1,*] & Arumugam Manthiram[3]

Affiliations:

[1]College of Chemistry, Zhengzhou University, Zhengzhou 450001, P. R. China

[2]Department of Mechanical Engineering, The Pennsylvania State University, University Park, PA 16802, USA

[3]Materials Science & Engineering Program, Texas Materials Institute, The University of Texas at Austin, Austin, TX, 78712 USA

[4]These authors contributed equally: Wei Guo, Wanying Zhang

Correspondence and requests for materials should be addressed to Y. Fu (email: yfu@zzu.edu.cn).

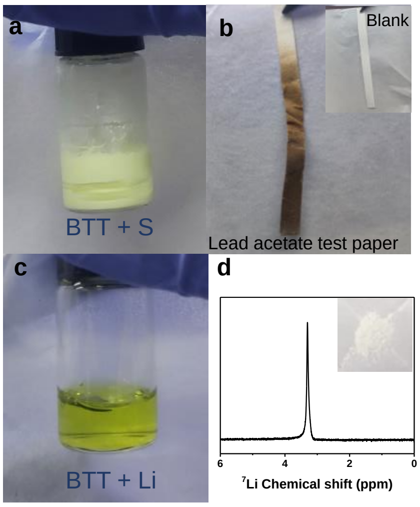

Supplementary Figure 1. **Magnification experiments for the reactions between BTT and sulfur or lithium. a** 50 mg BTT and 92 mg S powder were added as mole ratio 1:10 to the DOL/DME (1:1 v/v) solution. **b** Images for the lead acetate test paper in the vial and the blank one. Paper in the vial turns black meaning there is H_2S generated from **a**. **c** 50 mg BTT in DOL/DME solution and a piece of lithium foil was added into it, the solution becomes yellow and with little bubbles. **d** ^{7}Li NMR for the lithium benzenetrithiolate (Li_3-BTT) gained from **c**.

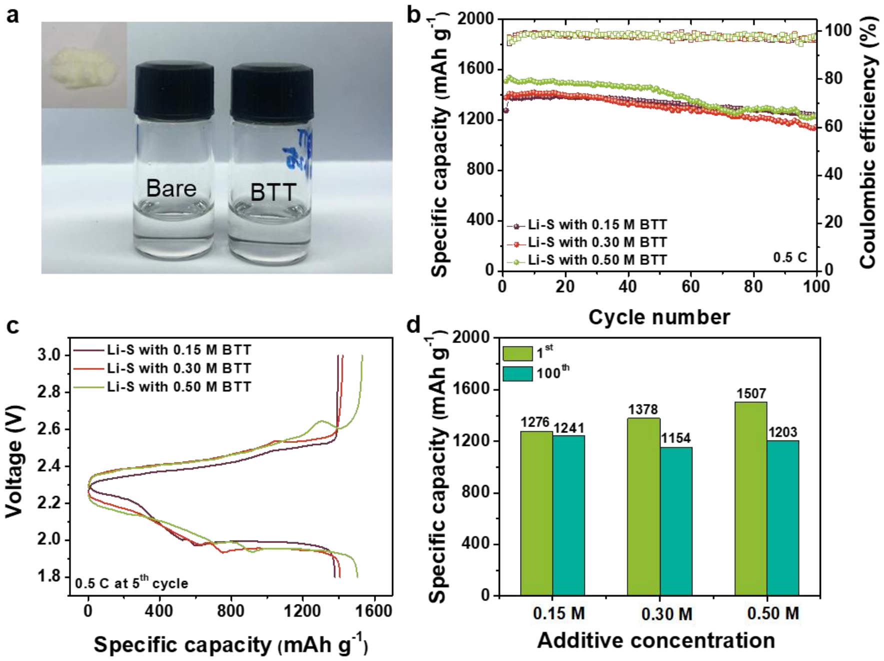

Supplementary Figure 2. Images for BTT powder and the electrolyte and electrochemical performance of Li-S cells with different concentration additive. a Images for the BTT white powder, BTT and blank electrolyte. **b** Cycling performance of Li-S cells with different concentration BTT at 0.5C rate**. c** Charge and discharge curves of Li-S cells with different concentration BTT at the 5th cycle. **d** Specific capacity of Li-S cells with different concentration BTT at the 1st cycle and 100th cycle.

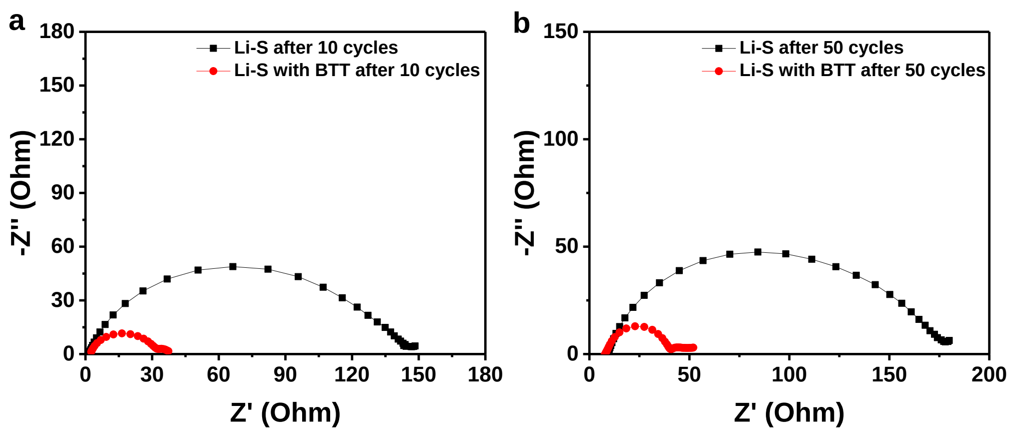

Supplementary Figure 3. Nyquist plots of the Li-S cells with BTT and blank electrolyte after cycle at 0.2C rate. a Two types of cells after 10 cycles. **b** Two types of cells after 50 cycles.

Supplementary Table 1. Impedance of the Li-S with BTT and blank electrolyte with different cycles, and the cells were cycled at 0.2C rate.

State	Li-S with BTT impedance at high frequency (Ω)	Li-S with BTT impedance at low frequency (Ω)	Li-S impedance (Ω)
Fresh	43	20	131
After 10 cycles	30	5	144
After 50 cycles	32	7	170

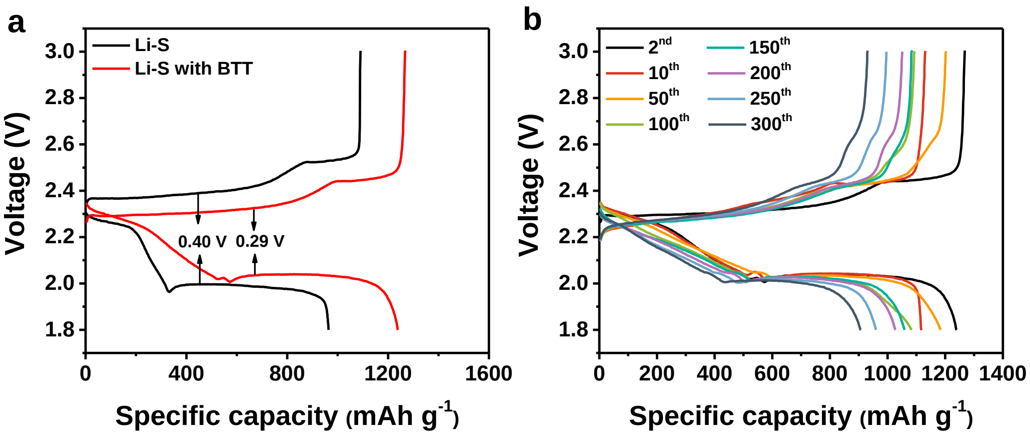

Supplementary Figure 4. Galvanostatic charge/discharge profile of Li-S cells. a The 2nd charge/discharge curves for Li-S cells with and without BTT at 1C rate. **b** Charge/discharge curves of different cycles for Li-S cell with BTT at 1C rate.

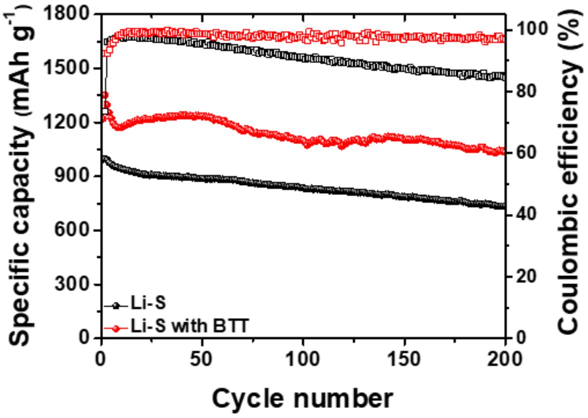

Supplementary Figure 5. Electrochemical performance of Li-S cells with BTT and blank electrolyte. Cycling performance of the two types of cells at 0.5C rate with 200 cycles.

Supplementary Table 2. Performance comparison of different electrolyte additives in Li-S cells.

Electrode	Electrolyte	Additive	Reversible capacity (mAh g^{-1})	Current (1 C = 1675 mA g^{-1})	Capacity retention	Ref.
Sulfur/carbon paper (S/MWCNT)	1 M LiTFSI 0.15 M $LiNO_3$ DOL/DME	1,3,5-Benzenetrithiol	907.3/300th cycle	1 C	87.6%	this work
Sulfur/carbon slurry	1 M LiTFSI TEGDME	P_2S_5	980/41th cycle	0.1 C	87.3%	28
Sulfur/porous carbon (S/PC)	1 M LiTFSI 0.4 M $LiNO_3$ DOL/DME	Pyrrole	607.3/300th cycle	1 C	53.5%	29
Sulfur/carbon black slurry	1 M LiTFSI TEGDME/DOL	Biphenyl-4,4′-dithiol	575/300th cycle	0.1 C	67.6%	41
Sulfur/carbon nanotubes (S/CNT)	1 M LiTFSI 0.3 M $LiNO_3$ DOL/DME	NH_4TFSI	340/200th cycle	0.1 C	37.1%	30
Hierarchical porous carbon (HPC)/S	1 M LiTFSI 0.15 M $LiNO_3$ DOL/DME	Dithiothreitol	471/500th cycle	0.5 C	58.3%	64
Sulfur/carbon nanotubes slurry	1 M LiTFSI 0.1 M $LiNO_3$ DOL/DME	Cobalt hexadecachloro phthalocyanine (CoPcCl)	803.9/200th cycle	0.3 C	73.4%	13
Sulfur/carbon black slurry	1 M LiTFSI 0.15 M $LiNO_3$ DOL/DME	Nitrogen-doped carbon dots (N-CDs)	447/600th cycle	1 C	59.6%	14
Sulfur/Ketjen Black carbon slurry	1 M LiTFSI 0.1 M $LiNO_3$ DOL/DME	Aluminum phosphate ($AlPO_4$)	665/100th cycle	0.2 C	76.0%	65
Sulfur/carbon black slurry	2 M LiTFSI 0.3 M $LiNO_3$ DOL/DME	Potassium Hexafluorophosphate (KPF_6)	942/100th cycle	0.2 C	77.8%	23

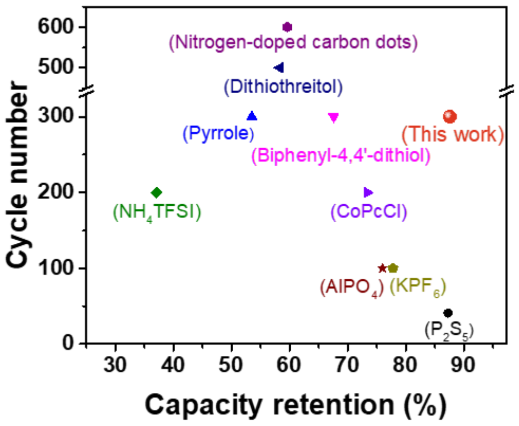

Supplementary Figure 6. Capacity retention and cycle number profile of different electrolyte additives in Li-S cells.

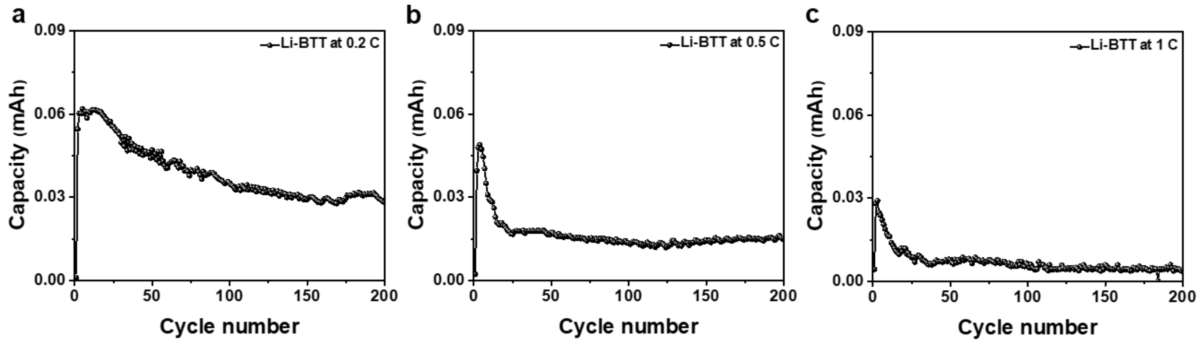

Supplementary Figure 7. Electrochemical performance of Li-BTT cell without active material. a b c Cycling performance Li-BTT cells at 0.2C, 0.5C, and 1C rate, respectively. BTT in the electrolyte just provides a little capacity and it decreases quickly after 30 cycles.

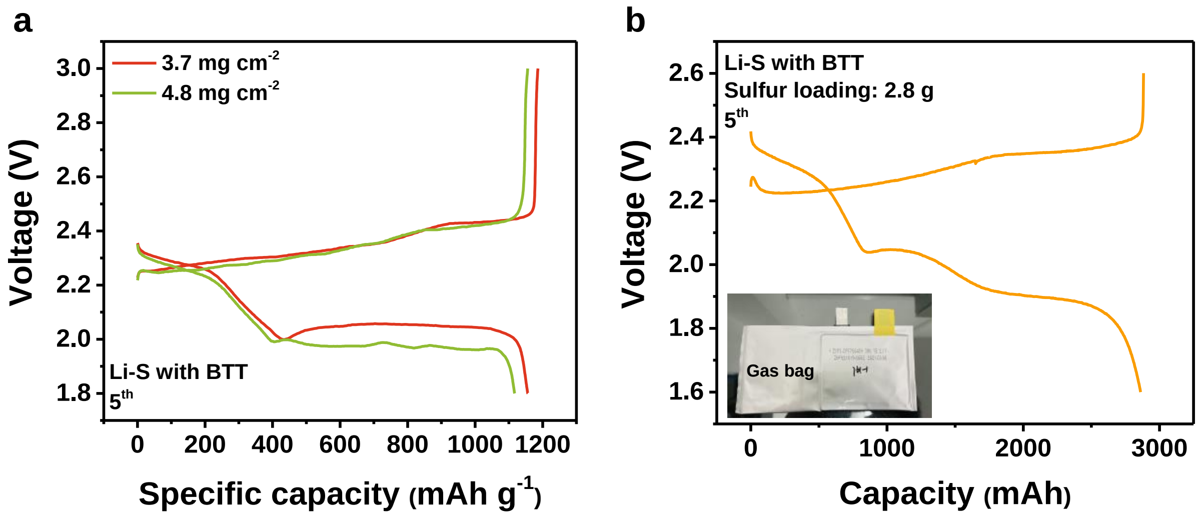

Supplementary Figure 8. Electrochemical performance of BTT cells with high loading. a Galvanostatic charge/discharge profile of BTT cells with high sulfur loadings at the 5th cycle. **b** Galvanostatic charge/discharge voltage profile of a Li-S pouch cell with BTT electrolyte in the 5th cycle, the total sulfur mass is 2.8 g and the E/S ratio is 2.6 μL mg^{-1}. The inset photograph shows the front image of the pouch cell with gas bag.

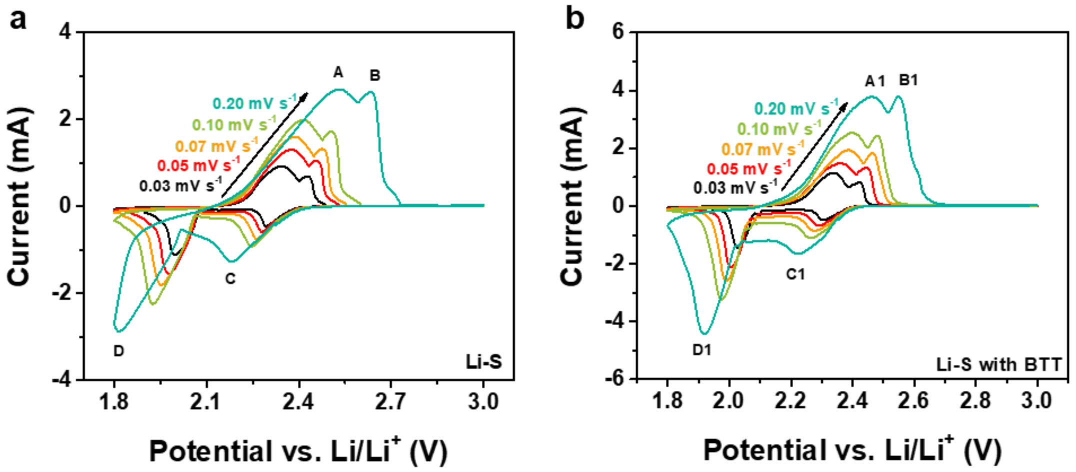

Supplementary Figure 9. CVs with different scan rates. a For Li-S cell and **b** For the Li-S cell with BTT electrolyte.

Supplementary Table 3. Lithium-ion diffusion coefficients calculated by the Randles-Sevcik equation from the linear fits of the peak currents.

Sample	D_{Li^+} (A/A1) cm^2s^{-1}	D_{Li^+} (B/B1) cm^2s^{-1}	D_{Li^+} (C/C1) cm^2s^{-1}	D_{Li^+} (D/D1) cm^2s^{-1}
Li-S	5.60E-08	6.78E-08	1.28E-08	5.49E-08
Li-S with BTT	1.32E-07	1.61E-07	2.51E-08	1.59E-07

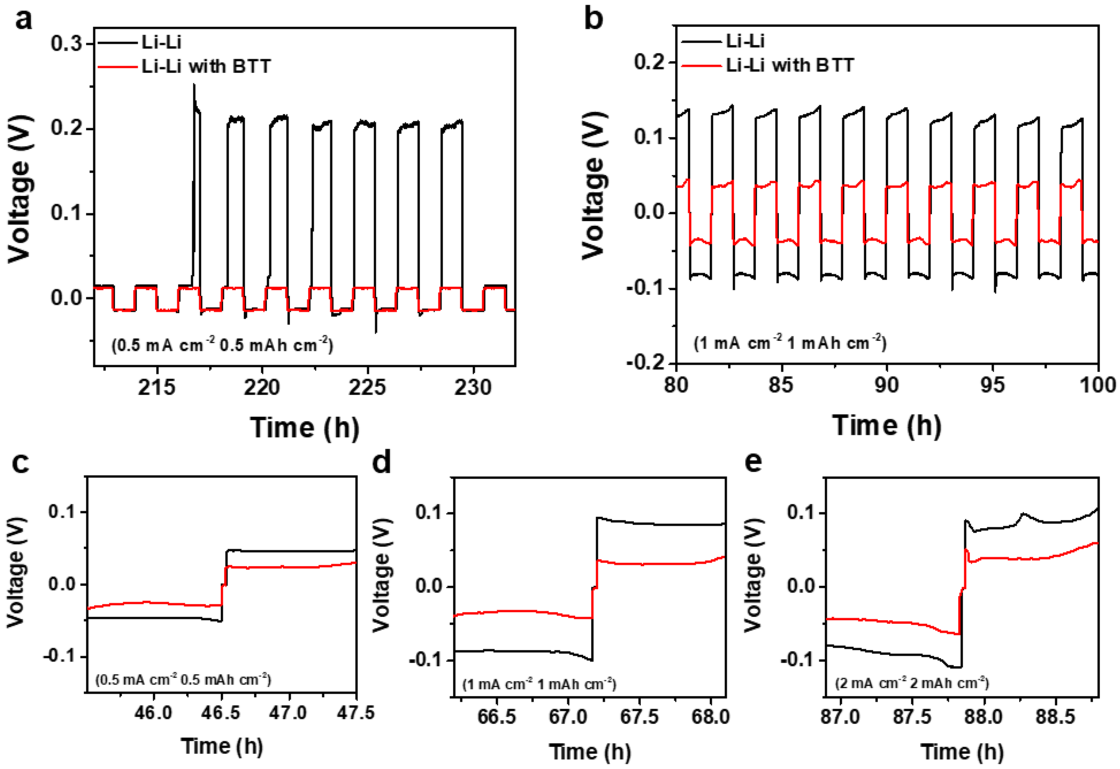

Supplementary Figure 10. Plating and stripping curves for the lithium symmetric cells with BTT and blank electrolyte. a Enlarged profile of two cells at 0.5 mA cm^{-2} (0.5 mAh cm^{-2}) for 10 cycles. **b** Enlarged profile of two cells at 1 mA cm^{-2} (1 mAh cm^{-2}) for 10 cycles. **c, d, e** Enlarged profiles of two types of cells at different current densities: 0.5 mA cm^{-2} (0.5 mAh cm^{-2}), 1 mA cm^{-2} (1 mAh cm^{-2}), and 2 mA cm^{-2} (2 mAh cm^{-2}).

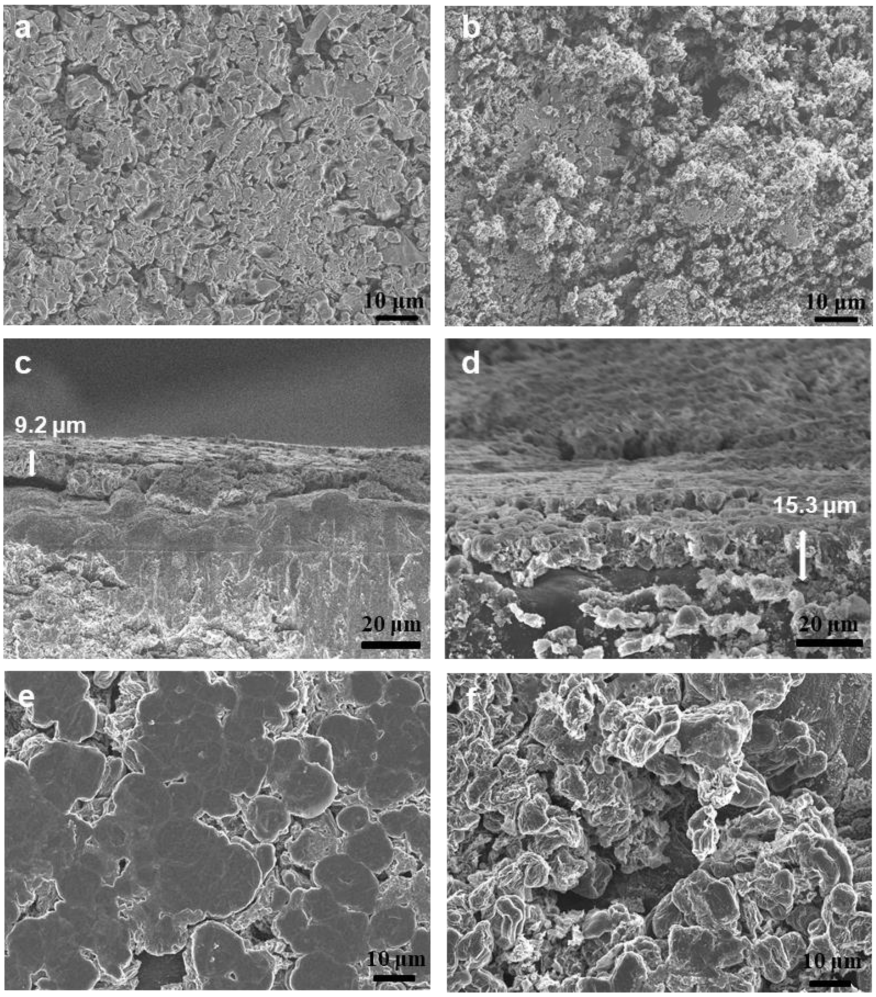

Supplementary Figure 11. SEM images for lithium foils in symmetric cells and Li-S cells. **a** Top view for the symmetric cell with BTT electrolyte after 100 cycles with current density of 0.5 mA cm^{-2} capacity density of 0.5 mAh cm^{-2}. **b** Top view for the symmetric cell with blank electrolyte. **c d** Cross view for the symmetric cells with BTT and blank electrolyte, respectively. **e f** Top view for lithium foils in Li-S cells with BTT and blank electrolyte after 50 cycles at 0.5C, respectively.

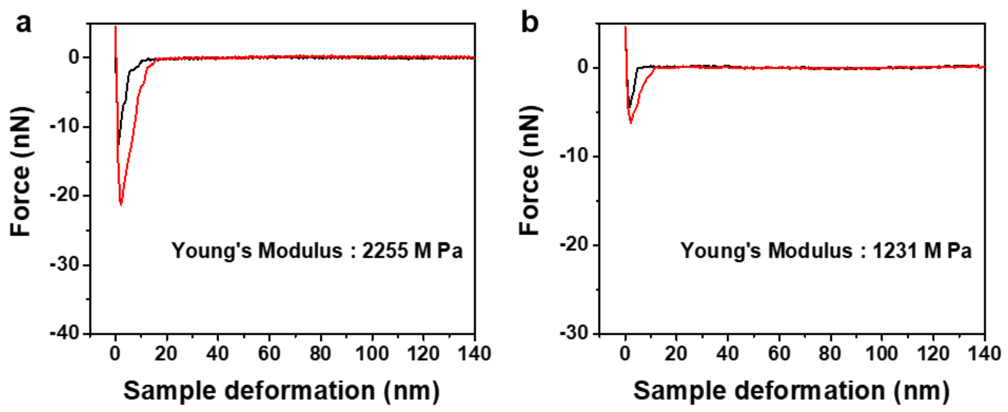

Supplementary Figure 12. Indentation curves of the SEI layer for the symmetric cells. a Profile of the SEI layer for symmetric cell with BTT electrolyte. **b** Profile of the SEI layer for symmetric cell with blank electrolyte.

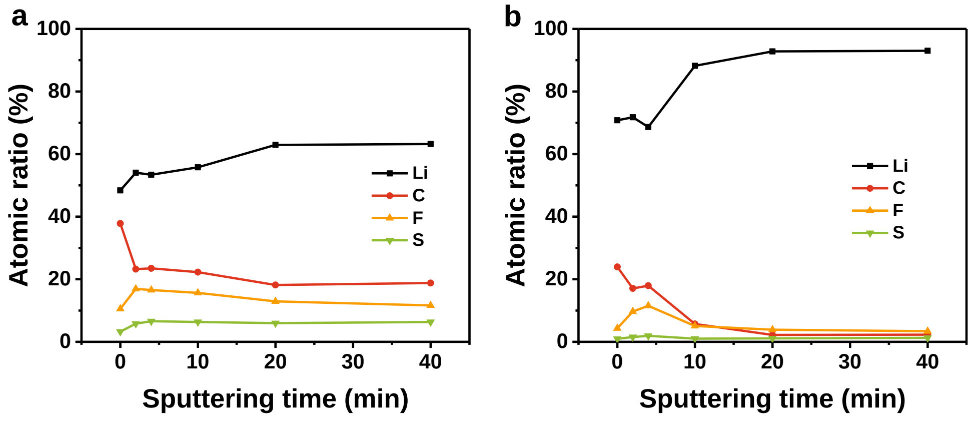

Supplementary Figure 13. Atomic percentage at various sputtering time. a Atomic ratio of the four elements in various sputtering depths for the SEI layer formed in lithium symmetric cell with BTT electrolyte. **b** Atomic ratio of the SEI layer formed in blank electrolyte.

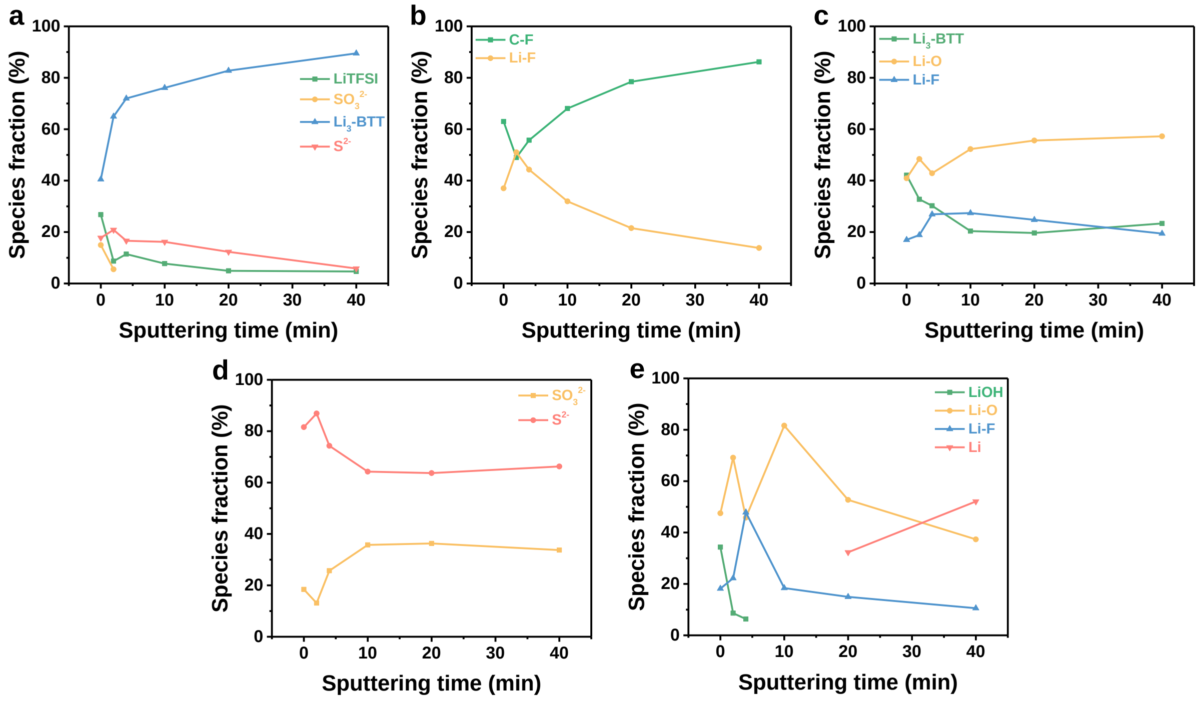

Supplementary Figure 14. Species fraction at various sputtering time. a b c Species ratio of the elements S, F, Li in different sputtering depths for the SEI layer formed in lithium symmetric cell with BTT electrolyte. **d e** Species ratio of the elements S and Li for the SEI layer formed in blank electrolyte.

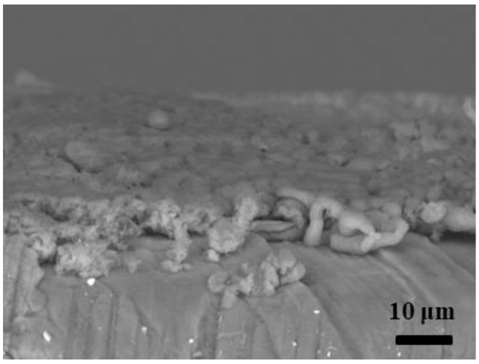

Supplementary Figure 15. Cross view SEM image for the lithium foil. Symmetric cell with BTT electrolyte after 50 cycles with current density of 0.5 mA cm^{-2} and capacity density of 0.5 mAh cm^{-2}.

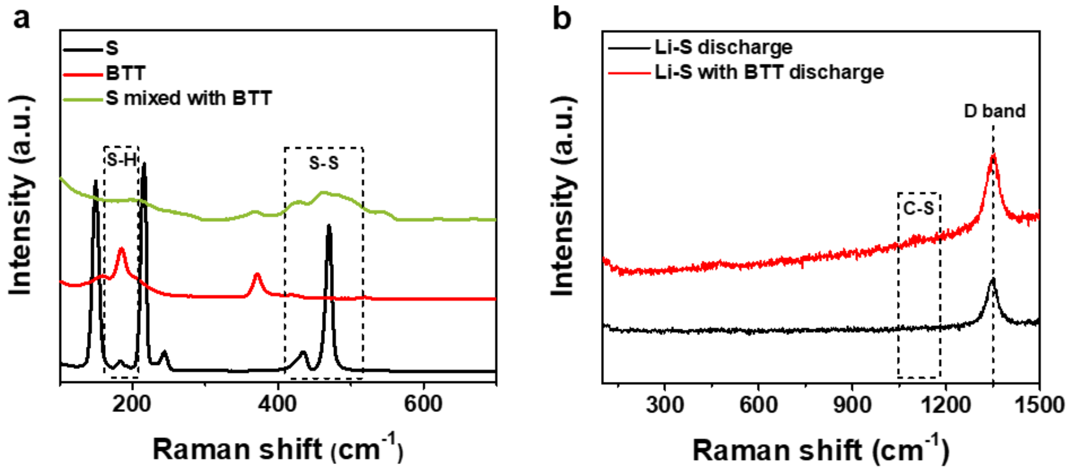

Supplementary Figure 16. Raman spectra. a Spectra of the raw material and the sulfur cathode with 0.15 M BTT electrolyte. **b** Spectra of the discharged cathodes for the Li-S cells with BTT and blank electrolyte.

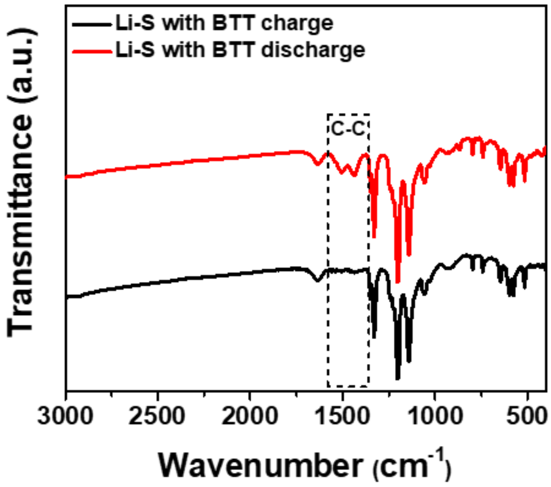

Supplementary Figure 17. FTIR spectra of the charged and discharged cathodes for Li-S cells with BTT electrolyte. There is a notable change of the C-C bond vibration for benzene ring.

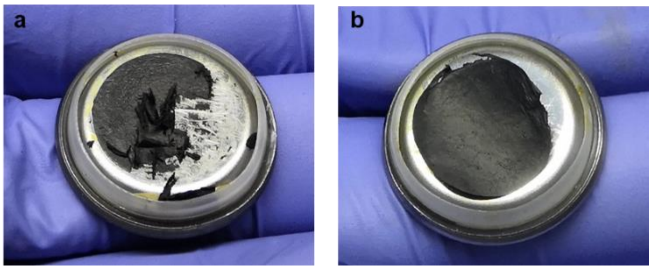

Supplementary Figure 18. Photographs of the recharged cathodes of Li-S cells with BTT and blank electrolyte at 0.2C rate. a Image for BTT cell. **b** Image for control cell.

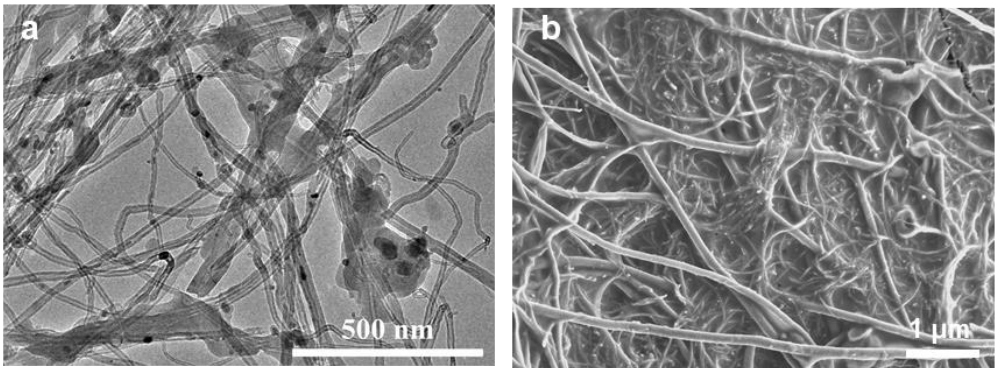

Supplementary Figure 19. **SEM and TEM images for the cathodes of BTT cells. a** TEM image of the recharged cathode for BTT cell with a 500 nm scale bar. **b** SEM image for the recharged cathode for the Li-S with BTT electrolyte after 10 cycles at 0.05C rate.

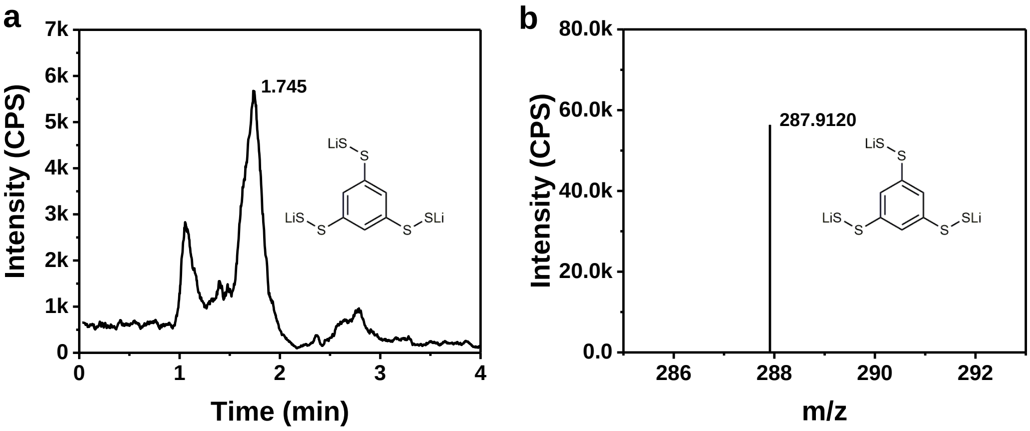

Supplementary Figure 20. **Incompletely discharge product (PhS_6Li_3). a** XIC of the discharge product for BTT cell. **b** MS profile and corresponding structure were inset.

Supplementary Table 4. Binding energies for Li 1s, F 1s, S 2p spectrum in the XPS spectra.

	Species	Binding Energy (eV)
Li 1*s*	Li-F	57.6
	Li-O	56.4
	Li_3-BTT	55.1
	LiOH	55
	Li	55.2
F 1*s*	C-F	687.3
	Li-F	685
S 2$p_{3/2}$	LiTFSI	169.2
	SO_3^{2-}	167.2
	S-S	164.1
	C-S	163.5
	Li_3-BTT	162.7
	S_2^{2-}	161.7
	S^{2-}	160.2

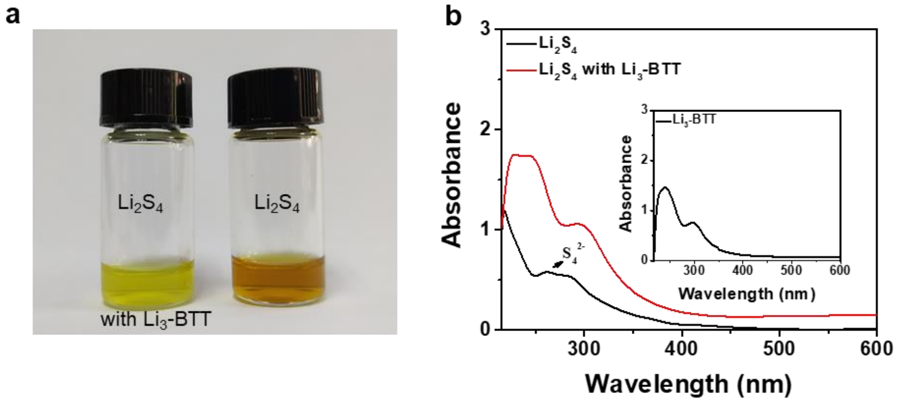

Supplementary Figure 21. Polysulfide solution in DOL/DME (1:1 v/v) and the reaction with Li_3-BTT. a Photograph image of the 5 mmol Li_2S_4 solution and the Li_2S_4 solution with 4 mg Li_3-BTT. **b** Corresponding UV-Vis spectra of the two solutions and the spectrum of blank Li_3-BTT dissolve in DOL/DME was inset.
